# Volume-outcome correlation in adrenal surgery—an ESES consensus statement

**DOI:** 10.1007/s00423-019-01827-5

**Published:** 2019-11-07

**Authors:** Radu Mihai, Gianluca Donatini, Oscar Vidal, Laurent Brunaud

**Affiliations:** 1 Churchill Cancer Centre, Oxford University NHS Hospitals Foundation Trust, Oxford, UK; 2 Department of Surgery and INSERM U1082, CHU Poitiers, University of Poitiers, Poitiers, France; 3 ICMDiM, Hospital Clinic, IDIBAPS, Universitat de Barcelona, Barcelona, Spain; 4 Department of Surgery and INSERM U954, CHU Nancy (Brabois), Université de Lorraine, Vandoeuvre les Nancy, France

**Keywords:** Adrenalectomy, Volume-outcome, Learning curve

## Abstract

**Background:**

Published data in the last decade showed that a majority of adrenal operations are done by surgeons performing only one such case per year and based on the distribution of personal workloads ‘high-volume’ surgeons are defined as those doing 4 or more cases/year.

**Purpose:**

This paper summarises literature data identified by a working group established by the European Society of Endocrine Surgeons (ESES). The findings were discussed during ESES-2019 conference and members agreed on a consensus statement.

**Results:**

The annual of adrenal operations performed yearly in individual countries was reported to be 800/year in UK and over 1600/year in France. The learning curve of an individual surgeon undertaking laparoscopic, retroperitoneoscopic or robotic adrenalectomy is estimated to be 20–40 cases. Preoperative morbidity and length of stay are more favourable in high-volume centres.

**Conclusion:**

The main recommendations are that adrenal surgery should continue only in centres performing at least 6 cases per year, surgery for adrenocortical cancer should be restricted to centres performing at least 12 adrenal operations per year, and an integrated multidisciplinary team should be established in all such centres. Clinical information regarding adrenalectomies should be recorded prospectively and contribution to the established EUROCRINE and ENSAT databases is strongly encouraged. Surgeons wishing to develop expertise in this field should seek mentorship and further training from established adrenal surgeons.

Adrenal surgery is a critical component of a service delivering comprehensive endocrine surgical care. The low incidence of adrenal pathology creates specific challenges related to the low-volume workload encountered in many hospitals. While in most countries there are many surgeons who perform in excess of 100 thyroid operations each year, only a small number of surgeons would perform more than 100 adrenal operations throughout their entire career. Centralisation of adrenal surgery is currently being discussed by different professional or governmental bodies. In the absence of significant changes in referral patterns and health care funding policies, individual clinicians have minimal opportunity to increase their personal experience and workload.

The aim of the paper is to review the published data demonstrating a relation between workload volume and outcome in adrenal surgery and to propose guidelines for future delivery of adrenal surgery.

## Incidence of adrenal disease in the population

### Adrenal Incidentalomas

Adrenal Incidentalomas (AI) are common within the general population. Due to the wide availability of high-resolution CT scanners, the prevalence of AI has increased from the 1–2% of older series to current estimates of 5–7% [1, 2]. Autopsy studies suggest a prevalence of clinically unapparent adrenal masses of around 2% (range 1.0–8.7%) [3]. Majority of AI are hormonally silent but some are found to be associated with catecholamines-, cortisol-, or aldosterone-excess secretion. The European Society of Endocrinology in collaboration with the European Network for Study of Adrenal Tumours (ENSAT) published extensive guidelines for the diagnosis and management of adrenal incidentalomas [3].

### Cushing’s disease

An incidence of 0.7/million/year was reported in northern Italy while a similar study in a single province in Spain reported a much higher incidence of 39 cases per million inhabitants [4]. This great variability might be explained by the heterogeneity of inclusion’s criteria used by different authors. A large multicentre observational study of 481 patients from 36 centres in 23 European countries showed that unilateral adrenal tumours were responsible for 27% of the whole cohort [5–7].

### Primary hyperaldosteronism and Conn’s syndrome

Historically primary hyperaldosteronism was considered a very rare (< 2%) cause of hypertension. In contrast, two recent meta-analyses reported a prevalence between 2.6 and 14.4% (mean value of 6%) when patients were evaluated in a referral centre [8] and even up to 27% (weighed mean value of 7.8%) [9, 10]. Such high estimates were confirmed in a meta-regression analysis on 42510 patients [2]. In parallel with a 9-fold higher odds for the prevalence in studies published after or before 2000, these values varies widely between reports from Australia, USA, and Asia vs. Europe (5.5-fold, 5.0-fold, and 1.5-fold higher prevalence compared with Europe, respectively) [2].

### Phaeochromocytoma/paraganglioma

The incidence of phaeochromocytoma (PHAEO) in the general population is 1–8 per million/year [11–13] and PHAEOs represent up to 5% of all benign adrenal masses. Autopsy studies reported undiagnosed PHAEOs in up to 0.1% patients [14]. PHAEOs have a strong association with genetically inherited conditions, with over 20 different susceptibility genes currently considered to be present in up to 30–50% of patients [15].

In summary, there is no significant variance in incidence and prevalence of benign adrenal tumours worldwide hence all countries face similar challenges related to service-delivery for the care of patients with relatively rare tumours. Particular issues related to adrenocortical cancer are outside the focus of this paper and have been detailed in several recent reviews and guidelines [16, 17].

## Who performs adrenal surgery

This issue has been addressed predominantly in the USA. Linderman et al. analysed the New York SPARCS database of 6.054 adrenalectomies and found that urologists (UR) performed more adrenalectomies (47%) than general surgeons (GS) (35%) and endocrine surgeons (ES) (18%) [18]. ES who performed adrenalectomies were more likely to be high-volume surgeons compared with GS or UR [18]. In contrast, Park et al. analysed 3.144 adrenalectomies performed between 1999 and 2005 and found that UR performed only 37% of operations while non-UR operated on 2.253 patients (73%) [19]. Another study from the USA showed that the majority of procedures were performed by non-UR (without distinction ES/GS) for both benign (57% vs. 43%) and malignant disease (66% vs. 34%), with no observed differences between the two specialties regarding morbidity and mortality [20]. In 2016, Sood et al. also showed that the large majority of adrenalectomies within the USA was performed rather by GS/ES than by UR both for benign (4.368 vs. 187) and malignant disease (268 vs. 21) [21]. In the largest study to date (58,948 adrenalectomies), Monn et al. showed that UR performed more adrenalectomy than GS (60% vs. 40%, respectively) but the percentage of adrenalectomies performed by UR has slowly decreased over time [22].

There are three reports from Europe on this topic. In 2008, the national Spanish database registered 1.042 adrenalectomies but unfortunately no distinction by specialty was available [23]. In 2016, Palazzo et al. reviewed hospital admissions the UK for the year 2013–2014 of 795 adrenalectomies. Urologists represented 19% (7 out of 36) high-volume surgeons (threshold ≥ 6 procedures/year) and 43% (63/146) of low-volume surgeons [24].

Overall, the published data show that surgical sub-specialty has no significant impact on perioperative outcomes and rather focus the debate on the positive correlation between increased surgical expertise (as estimated by volume workload) and perioperative outcomes.

## What is the individual workload of adrenal surgery reported in the literature

There is a plethora of papers reporting initial experience, extended experience, and multi-institutional, national, or international collaborations. Some results extrapolated from national surveys or registries may harbour some limitations and biases.

The national audit of the British Association of Thyroid and Endocrine Surgeons (BAETS) reported 2.073 adrenalectomies between 2012 and 2017 [25] but disclosure was on voluntary bases and the audit captured only a fraction of the cases as there are 7–800 adrenalectomies performed yearly in the UK [24]. The individual annual workload of 50 surgeons who reported their own data ranged between 1 and 40/year [25]. Data from Netherlands does not provide an individual workload but emphasises that those working in a hospital recognised by the Danish Adrenal Network have better outcomes [26].

In France, 1681 adrenalectomies were performed in 2017 and university hospitals performed the majority of them in 25 high-volume centres (all academic, almost all with a specialised unit in endocrine surgery) with a mean workload of 42 adrenalectomies/year. The rest of 229 public and private hospitals shared the remaining 627 adrenalectomies (mean value = 2.8 adrenalectomies/year). This trend between university and teaching hospitals and small peripheral hospitals had become evident since the early 2000 [27]. In a more recent analysis of 9820 patients operated in France between 2012 and 2017, 280 centres had performed five or fewer adrenalectomies during the 6-year period of the study and only 26 centres performed half of the national instances of adrenalectomy [28].

At the top range of number of adrenalectomies performed in the same institution, one can find reports of 560 retroperitoneoscopic adrenalectomies or over 300 robotic adrenalectomies [29, 30] or 420 adrenalectomies during 19 years and 520 patients over 20 years [31, 32]. A recent meta-analysis that reviewed 29 series from endocrine referral centres comparing different surgical approaches for adrenalectomy included reports of 10 up to 267 cases [33]. Due to the extreme rarity of the disease, large series adrenocortical carcinomas have reported only through multi-institutional cohorts studies (e.g. the report by Lee et al. on 201 patients with adrenocortical cancer [34]).

Low-volume series are also found in the literature predominantly from developing countries where centralisation might be scarce hence the authors reported 34 adrenalectomies in 10 years in Saudi Arabia (a mean of 3/year) [35], or 46 adrenalectomies in 6 years in Thailand (7.6/year) [36] or 35 adrenalectomies over 12 years in three different hospitals (1 adrenalectomy/year/hospital) [37].

## Published evidence for a learning curve

The literature about the learning curve for minimally invasive adrenalectomy is scarce. Few small controlled trials are available and most studies are retrospective in nature, with significant heterogeneity among them and increased risk for publication bias as well as other confounding factors. In addition, reporting of outcomes and follow-up varies significantly, making it difficult to combine and compare such data. Finally, most of the studies do not report details on the previous expertise of their surgeons making the generalisation of their findings difficult [38].

### Laparoscopic adrenalectomy

Laparoscopic adrenalectomy has been performed since the early 1990s and has shown favourable results over open abdominal surgery, including decreased blood loss, postoperative pain and complications, and shorter hospital stay [39–41].

The laparoscopic transperitoneal approach is most frequently performed and is considered to be the standard procedure for adrenalectomy. Studies from high-volume centres with experienced endocrine surgeons have demonstrated that approximately 20–40 procedures are required to overcome the learning curve [42, 43]. Goiten et al. assessed the outcome and learning curve of the first 100 cases operated by the same surgical team in a retrospective analysis of prospectively collected data. In order to assess the learning curve, procedures were divided into three, equal consecutive groups (*n* = 33, 33, and 34). Intraoperative complications in the intermediate and late groups were significantly less compared with those in the early group (2/33, 2/34, and 7/33, respectively; *P* < 0.05). Similarly, the mean operating time was significantly reduced between the early (169 min) and both intermediate (116 min) and late (127 min) groups (*P* < 0.005). The conversion rate was reduced between the three groups (3/33 vs. 2/33 vs. 0/34), but this was not significant. They concluded that the performance of approximately 30 cases by an experienced laparoscopic surgeon is required to master the procedure [42]. Similarly, Sommerey et al. evaluated the surgical outcome of 215 laparoscopic adrenalectomies performed over a 10-year period demonstrating that for surgeons unfamiliar with the technique is mandatory to give intensive train with a defined plan to accomplish the learning curve before the start of technique without supervision [43].

Current SAGES guidelines for minimally invasive treatment of adrenal pathology recommend that dedicated, advanced training should be pursued by surgeons unfamiliar with this technique. Until proficiency with LA is attained, referral to a centre with expertise in minimally invasive adrenal surgery should be considered [38].

### Retroperitoneoscopic adrenalectomy

The minimally invasive retroperitoneal approach for adrenalectomy (RA) has gained popularity since its introduction and standardisation by Martin Walz [44]. This approach offers a more direct route to the adrenal glands, is feasible in obese patients and in those with a history of abdominal surgery, and avoids repositionining during bilateral adrenalectomy. Despite several published reports showing shorter duration of surgery, less blood loss, decreased postoperative pain, faster recovery, and improved cost-effectiveness with RA, many surgeons are reluctant to embrace this technique.

The length of the learning curve for RA has been assessed in a multicentre prospective study from the Netherlands, Australia, and Sweden [45]. The first 50 consecutive RA performed by individual surgeons and their teams were included in the analysis. A total of 181 surgical procedures performed by four surgical teams were analysed. The median duration of operation was 89 (range 29–265) min. The learning curve cumulative sum analysis showed that competency was achieved after a range of 24–42 procedures, with significant difference between individual surgeons.

Similarly, a short learning curve was seen in 113 RA performed in a single centre for a single surgeon with extensive experience in laparoscopic adrenal surgery: the operating times decreased significantly from a median 100 min in the first 20 patients to 60 min after 40 patients [46].

As RA is being usually indicated only in a minority of patients (small tumours < 4–5 cm), it might take several years before a surgeon performs enough operations to have completed his/her learning curve and this might explain the reluctance to introduce RA in a surgical unit with low/moderate annual workload.

Overall the learning curve for LA and RA appears to be 20–45 cases. In general practice, this number may be difficult to achieve due to the rarity of these procedures. Controversy exists as to which approach, anterior or posterior, requires more cases for the surgeon operative time and the patient morbidity to plateau. However, learning curve comparisons between different surgical approaches are difficult because other factors such as the surgeon previous experience and the operative team familiarity and pre-implementation training also significantly influence the procedure learning curve.

### Robotic adrenalectomy

In 1999, Piazza et al. published the first case of robot-assisted right adrenalectomy in a patient with Conn’s syndrome using the ZEUS AESOP (Computer Motion, Inc., Santa Barbara, CA) [47]. After the introduction of the da Vinci system (Intuitive Surgical, Sunnyvale, CA, USA), several series of robotic surgery have been reported.

Enthusiasts who embraced this technique emphasise its perceived benefits with 3D vision, the elimination of surgeon tremor and increased degrees of freedom of the surgical instruments, and a comfortable operating position but have to acknowledge the longer operative time and more expensive costs compared with traditional laparoscopic surgery. Keeping the robot in a dedicated operating room, completing all preparations of the robotic platform during induction of anaesthesia, and using a surgical team familiar with robotic surgery can reduce the operative time. The learning curve is certainly affected by previous exposure to other robotic operations. Brunaud et al. observed no significant differences in operative time after the learning curve of 20 cases [48] while Agcouglu et al. reported a significant improvement in operative time after only the procedure number 10 in the robot-assisted group [49].

A meta-analysis comparing RtA and LA identified 13 studies including 798 patients. Three hundred seventy-nine underwent RtA (cases group) and 419 LA (controls group). Eight studies were prospective, but only one was a randomised controlled trial and five studies were retrospective. Overall complications and conversion rate seemed to be favouring of RtA but with no significant differences, despite a significant lower length of hospital stay after robotic surgery. There was a significant reduction of estimated blood loss in RtA due to stereoscopic vision and to more precise dissection plane when using the robotic arms. Although this difference was statistically significant, it was probably not clinically relevant. There were no significant differences in terms of conversion and overall complications rate [50].

### Open radical adrenalectomy

There is no published data regarding the learning curve for this complex operation currently reserved to malignant tumours or very large (> 10 cm) phaeochromocytomas. It is expected that surgeons offering to undertake this procedure would have significant previous experience with laparoscopic adrenalectomy or other major complex intrabdominal oncological operation. Issue related to selection for open vs. laparoscopic operation and the requirements for open adrenalectomy have been discussed in guidelines issued by ESES and ENSAT [51].

## Is there evidence that complication rates or length of stay varies with workload?

Adrenalectomy has a low mortality rate. The BAETS 2017 national audit report identified 9 deaths during the index admission out of 1840 adrenalectomies registered (0.5% mortality rate) but no correlation was made with the workload of individual surgeons and no cross-checks made with other hospitals records for these self-declared events [25]. The French study analysed 162 cases of mortality out of 9820 adrenalectomies, with a mortality rate of 1.0 % in 254 low-volume centres and 0.4% in 12 high-volume centres. At 90 days, the mortality rate was 1.8% in low-volume centres and 0.9% in high-volume centres (*P* < 0.001) [28]. Similarly, in the study by Park et al. of 3144 adrenalectomies, high-volume surgeons had a significantly lower mortality rate of (0.56% vs. 1.25%, *P* = .004) [18]. It is important to note that this mortality rate is twice as high as after bariatric surgery hence this aspect cannot be ignored and surgeons with low volume and limited experience should use a risk stratification in order to choose patients who might benefit from referral to a regional centre with larger experience.

Postoperative in-hospital complication rate of 20% and variable hospital durations of stay of 2–9 days have been reported. A number of studies have assessed the impact of both surgeon and hospital volumes on postoperative outcomes for adrenalectomy. While all report an association between treatment by either a high-volume surgeon or at a high-volume hospital and improved outcomes, the method of defining a high- vs. low-volume surgeon varied across the studies. Volume thresholds are often selected arbitrarily or based on quartiles or quintiles, which makes comparison across studies difficult [52]. In the study by Park et al. of 3144 adrenalectomies performed between 1999 and 2005, adrenalectomy by low-volume surgeons (< 4 cases/year) was associated with more complications (18.3 vs. 11.3% for high-volume surgeons, respectively, *P* < 0.001) and longer durations of stay (5.5 vs. 3.9 days for high-volume, *P* < 0.001) [19]. In 2018, Lindeman et al. confirmed those data and showed that high-volume surgeons had a significantly lower complication rate (14.4% vs. 8.8% *P* < .001) and lower median LOS (high volume: 2 days [IQR 1–5] vs. low volume: 4 days [IQR 2–7], *P* < .001) [18].

Anderson et al. performed a retrospective cohort study of hospital discharges of 6712 patients operated by 3496 surgeons who performed adrenalectomies at 687 hospitals from the USA between 1998 and 2009 [53]. Overall 1378 (20.5%) patients experienced at least one complication after adrenalectomy. After adjusting for patient demographic, clinical, and hospital variables, increasing annual surgeon volume was associated with decreasing odds of experiencing a postoperative complication up to 6 cases per year. Based on the threshold ascertained from this analysis, high-volume surgeons were categorised as those performing ≥ 6 adrenalectomies per year. Patients who had their adrenalectomy completed by a low-volume surgeon were the majority (83%), were more likely to be older (60 vs. 56 years, respectively), of black race (11% vs. 7%), and covered by government-provided health insurance (Medicare and Medicaid; 48% vs. 37%) in a nonteaching (40% vs. 5%) hospital. They were also more likely to have a urologic complication (5% vs. 2%, respectively, *P* < .001), respiratory complication (7% vs. 5%, *P* = .008) or any complication (22% vs. 14%, *P* < .001) and they had higher rate of in-hospital mortality (2.4% vs. 0.6%, *P* < .001). Their hospital duration of stay was greater (median 6 vs. 3 days, respectively, *P* < .001) and the cost of their care was higher, with a difference of $1,659 per patient (median $11,543 vs. $9884, *P* < .001). Compared with the patients of high-volume surgeons, patients of low-volume surgeons had a 36% increase in the odds of having a complication if the surgeon performed 1 case per year, 24% for 2 cases per year, 15% for 3 cases per year, 8% for 4 cases per year, and 3% for 5 cases per year (all *P* < .05) [53].

Using the same threshold cut-off point of 6 adrenalectomies/year applied to data retrieved from Hospital Episode Statistics in England, Palazzo et al. found that hospital duration of stay was 60% greater and rates of 30-day readmissions were 47% greater for low- vs. high-volume adrenal surgeons [24].

Despite their limitations, all these studies suggest that a significant improvement in outcomes can be observed at a threshold of 6 cases/year. This model could be tested in the future using a prospective database recording details of surgery, comorbidities, and 30-day complication rates stratified on severity (e.g. Clavien scale).

## Who reports rare complications after adrenalectomy?

It is impossible to show whether unusual or very severe complications occur in patients treated in low-volume centres. Such data does not exist in the literature. Reports about significant intraoperative vascular lesions address problem solving during minimal invasive procedures. For example, Morelli et al. reported two vascular lesions (one vena cava and one left renal vein damage) among intraoperative complications in RtA for large adrenal tumours managed by using sutures without the necessity to convert to laparoscopic or open surgery [54]. Bleeding has been the most common reported complication of adrenalectomy. However, a number of high-grade complications of adrenalectomy not typically reported in the literature have come to light in the minimally invasive era including injuries to the porta hepatis that have resulted in liver failure and have led to liver transplantation, injuries to the renal vessels or ureter requiring nephrectomy, and inappropriate organ resection, particularly mistaken resection of the tail of the pancreas instead of the adrenal gland [55].

## What parameter can be used to assess/define expertise in adrenal surgery ?

### Tumour diameter

Laparoscopic adrenalectomy (LA) was initially adopted to treat small benign tumours but nowadays it is considered the “gold standard” technique to treat a broad spectrum of functioning and non-functioning adrenal tumours up to 12–15 cm. Most authors consider the tumour dimension > 12 cm a contraindication to LA [56] but several recent studies indicated that LA might be performed safely even for masses up to 15 cm [57–59]. Indications to LA for lesions > 6 cm is a matter of debate and experienced endocrine surgeons are divided between supporters [60] and detractors [61, 62] depending on their views about the risk of malignancy in such cases hence surgeons should be familiar with the current recommendations of ESES and ENSAT [16, 17, 51].

For phaeochromocytomas, LA has been described as an effective and safe approach even for tumours > 6 cm in diameter but patients with such large tumours may have a higher conversion rate and more intraoperative hypertensive crises [63].

### Intraoperative complications

The most frequent intraoperative complications are bleeding from adrenal and renal vein or adrenal cortex, vena cava injuries, diaphragmatic perforation, and spleen laceration. Retroperitoneal hematoma, incisional hernia, pancreatic fistula, hyponatremia, and intestinal injuries are the most common postoperative complications [64]. Considering the complications rate after LA and RA is really important to evaluate several preoperative risk factors which could affect morbidity incidence. Many studies demonstrate that the size of the mass and the histopathological diagnosis of phaeochromocytoma were independent risks factors of the perioperative complications rate. LA in large adrenal masses (> 8 cm) is associated with prolonged operative time, increased blood loss, and longer hospitalisation, without affecting perioperative morbidity [65].

### Conversion rate

Obesity is an important risk factor affecting morbidity after LA. Erbil et al. showed a positive correlation between BMI and operating time, postoperative complications, and hospitalisation as the result of a suboptimal visualization in the context of increased amount of intraperitoneal fat [66]. Nevertheless, previous series did not lead to the same conclusions, considering obesity only as increasing the conversion rate and the operative time.

### Oncological outcome

Minimally invasive surgery of large adrenal tumours has to follow oncological principles such as sufficient resection margin, intact tumour capsule, no malignant cell dissemination, and no risk for port-site metastasis [67]. It is above the scope of this paper to comment about the relationship between surgical approach and oncological outcomes for adrenocortical cancer and specific guidance from ESE, ESES, and ENSAT should inform such decisions [16, 17].

Miller at al. reported a worse oncological outcome in patients who underwent LA vs. open surgery, with a shorter mean disease-free survival (9.6 months vs. 19.2 months) and a higher recurrence rate (35% vs. 28%), as confirmed also by other studies [68, 69]. A German study including 152 patients showed a comparable frequency of tumour capsule laceration and peritoneal carcinomatosis between laparoscopy and open surgery, with the advantage in the minimally invasive group of an enhanced quality of life [70].

A French study evaluating LA vs. open surgery for stage I/II adrenal cancer reported a Kaplan-Meier estimated 5-year disease-specific survival and disease-free survival identical in both groups (*P* = 0.65 and *P* = 0.96, respectively) [71]. Furthermore, a recent Italian paper underlined the importance of a multidisciplinary approach for adrenocortical cancer, achievable only in dedicated high-volume centres [72].

Robotic assistance has been rapidly adopted by urological surgeons and has become particularly popular for oncological procedures involving the retroperitoneal space. The wide dissemination of robot assistance probably reflects the limited amount of operating space available within the retroperitoneum and the advantages provided by robot-assisted approaches, including 3D imaging, wristed instrumentation, and the shorter learning curve compared with that associated with the equivalent laparoscopic techniques. Surgical procedures that have traditionally been performed using an open or laparoscopic approach, such as partial nephrectomy, radical nephrectomy, retroperitoneal lymph node dissection, nephroureterectomy, and adrenalectomy, are now often being performed using robot assistance. The frontiers of robot-assisted retroperitoneal oncological surgery are constantly expanding, with an emphasis on maintaining oncological and functional outcomes, while minimising the level of surgical invasiveness [73]. However, prospective studies on oncological outcome with long-term follow-up are mandatory before to reach any recommendation about oncological robotic adrenal resections.

Finally, while studies on the safety of any type of adrenalectomy often come from high-volume centres, national data show that the average surgeon who performs adrenalectomy only does one case on average per year. Given this discrepancy between the literature and the observed practice patterns, we suggest that agreeing on a minimum volume threshold for surgeons for undertake adrenalectomy is of highest importance.

## Should there be a minimum annual workload?

The question of the minimal annual workload has been discussed for many years of many surgical specialities and a large amount of literature has been published [74]. A Pubmed search using the keywords ‘operative volume’ and ‘adrenalectomy’ led to 40 papers.

A multicentre study from the New York Statewide Planning and Research Cooperative System from 2000–2014 included 2839 adrenalectomies performed by 462 urologists (47%), 1098 by 23 endocrine surgeons (18%), and 2117 by 599 general surgeons (35%). Median annual surgeon volume was 1 case (IQR 1–2) with a mean of 2.1 cases, and a range of 1 to 29 cases. This emphasises that most of adrenalectomies in this area were performed by surgeons doing less than 3 cases per year. This trend is likely similar in Europe and this was confirmed in one study from the UK [24] reporting 795 adult adrenalectomies performed by 222 different surgeons with a range of between 1 and 34 adrenalectomies performed per surgeon. Only thirty-six (16%) adrenal surgeons performed 6 or more adrenalectomies. A total of 186 surgeons (84%) performed a median of one adrenalectomy a year. Overall, this shows that the number of adrenalectomy per year and per surgeon is very low.

This low average number of cases per year and per surgeon also infers that it has been difficult to define a meaningful/relevant cut-off number associated with improved perioperative outcomes. Overall, there is no validated consensus to define minimum annual workload in relation with improved perioperative outcomes. However, data from two studies support a minimum annual workload of at least 6 adrenalectomies per surgeon and per year.

## Financial models of reimbursement and its impact on the feasibility of new techniques

There is a significant relationship between minimal annual workload and costs. As funding principles vary widely in the health care systems of different countries, direct comparison is impossible and extrapolation is not valid. It remains likely that financial models of reimbursement have an impact on the feasibility of a specific new technique and could influence the decision to adopt new techniques.

In a recent study, patients undergoing resection by low-volume surgeons (< 6 cases/year) had an increased cost (+ 26.2%, 95% confidence interval, 12.6–39.9, *P* = .02) [53]. Using data on 7045 patients from the Nationwide Inpatient Sample (2003–2009), some calculated that if all operations performed by low-volume surgeons (1 case/year) were selectively referred to intermediate-volume surgeons (2–5 cases/year), a 7.7% cost savings would have been incurred. Potential savings were even higher (8.1%) if the operations had been performed by the high-volume surgeons (≥ 7 cases/year). With the conservative assumption that there are 5000 adrenalectomies per year in the USA, the high-volume surgeons would produce savings of $8.8 million over a span of 14 years [75].

In a similar analysis of 6416 patients who underwent adrenalectomy in the Healthcare Cost and Utilization Project Nationwide Inpatient Sample (HCUP-NIS) from 2003 to 2008, patients 61–70 years (22% of the cohort) and > 70 years (13% of the cohort) had more complications (14% vs. 20% vs. 23%), longer mean length of stay (3.3 vs. 4.0 vs. 4.9 days; *P* < 0.001) and higher mean costs ($12,307 vs. $13,226 and $14,649; *P* < 0.001). As older age seems to be independently associated with adverse short-term clinical and economic outcomes after adrenalectomy, the authors suggested that enhanced access to high-volume surgeons is a potentially modifiable factor of particular importance in these patients [76].

The financial concerns related to delivery of adrenal surgery are more significant when considering robotic surgery. Widespread adoption of robotic technology has positioned robotic adrenalectomy as an option in some medical centres. Speculative advantages associated with the use of the robotic system have rarely been evaluated in clinical settings and cost increase remains an important drawback associated with robotic surgery. The higher cost of robotic adrenalectomy is a documented drawback of the procedure. It was calculated that robotic adrenalectomy was 2.3 times more expensive than laparoscopic adrenalectomy [47]. Increasing the number of robotic cases performed per year and depreciation of robotic system were more effective in cost reduction compared with decreasing operative time. Winter et al. calculated in the USA that if a centre performs over 500 robotic operations per year, then capital and maintenance costs for the robot would be $380 per procedure [77]. From this standpoint, it has been considered that robotic adrenalectomy could become more affordable in high-volume robotic surgery centres in the future [78].

In 2018, Feng Z et al. reviewed their experience and strategies to reduce the cost of robotic adrenalectomy (RtA) comparing it with laparoscopic adrenalectomy (LA). The calculated relative costs were similar: $3527 for RtA and $3430 for LA. The average consumables fees were $1106 for RA vs. $1009 for LA (*P* = 0.62). The average postoperative hospital stay was similar (1.7 days vs.1.9 days). It appears that limiting the number of robotic instruments and energy devices while utilising an experienced surgical team can keep the costs of RtA comparable with those of LA [79].

Overcosts due to the use of the robotic system could also be balanced by hospital stay decrease, patients' referral increase, improved postoperative outcomes in more difficult patients, and ergonomics for the surgeon. However, the current surgical intuitive business model is counterproductive, because there are no available strong clinical data that could balance overcosts associated with the use of the robotic system [80].

There is no available data published on the reimbursement of different adrenalectomy techniques (i.e. retroperitoneal, transperitoneal, robotic) from different health care providers. A comparison between different countries is not available. In France, robotic adrenalectomy has the same reimbursement than conventional laparoscopic adrenalectomy (flat reimbursement). The posterior approach and transperitoneal approach have also the same amount for cost reimbursement. Consequently, this flat reimbursement rate does not favour the use of new techniques (ex robotic platform) because they are often more expensive. Currently South Korea being the only country having a higher reimbursement rate for robotic surgery.

## Final comments about the benefits vs. risks when choosing the technique for adrenalectomy

All available techniques for adrenalectomy can be divided into two groups. The first one is open adrenalectomy and in 2018, this approach is (or should be) used only in patients with large tumours and/or with local extension. The decision of using laparotomy is made by necessity when resection of large adrenal tumours and/or with local extension is technically too difficult using a laparoscopic approach or when the laparoscopic approach leads potentially to decreased postoperative patients survival (e.g. capsular effraction). This decision is very subjective and depends upon personal experience and expertise. The threshold in terms of tumour diameter to define a large tumour is also not consensual and would be ranging from 6 to 12 cm (depending on experience, surgeon and first aid skill, collaborating endocrinologists, anesthesiologists, use of an updated laparoscopic or robotic platform). There is no risks/benefits balance to be performed when choosing an open approach because this choice is imposed by clinical factors specific to each patient.

The second group represents all minimally invasive approaches with two main options: laparoscopic transabdominal and laparoscopic posterior adrenalectomy. Perioperative results are similar between these two techniques with the exception that tumours larger than 6 cm are more difficult to be resected using the posterior approach. Risks vs. benefits evaluation for these two approaches are likely similar in patients with tumours < 6 cm. For larger tumours, the feasibility of transabdominal laparoscopic approach is likely superior.

The robotics system allows to perform both transperitoneal and posterior approaches. There is the same feasibility issue for posterior robotic approach (than conventional posterior laparoscopic approach) in patients with tumours > 7 cm. Conventional laparoscopy and robotic approaches have similar results in patients with usual tumours. The robotic system may be superior in patients with more complex and difficult cases (tumour > 8 cm, right side, need to control inferior vena cava, large phaeochromocytoma or paraganglioma). This remains to be validated in future studies

## Role of audit/reported outcomes/minimum datasets

The role of the European database EUROCRINE is crucial (www.eurocrine.eu). Eurocrine has been designed to evaluate activity and quality in endocrine surgery in each participating European country. The minimum dataset has been discussed extensively and is already running within the database. Audits have also been anticipated for this database. In the coming years, it is likely that this large dataset will facilitate significant studies in this field.

## Future research studies

There are significant uncertainties that could be addressed in multicentre cohort studies, such as the following:

- To evaluate the difference between minimum annual workload per surgeon vs. per medical centre. Even though physician volume is an important variable, surgery is a team effort and an effective team is crucial for communication, early identification of complications, and access to critical related services. Surgeon volumes may change when they join teams or hospitals with higher volume referrals for surgery. In this context, hospital volume instead of surgeon volume might be more relevant but this remains controversial [81].
- To investigate whether a higher threshold of adrenalectomies per surgeon and per year (i.e. > 6 vs. > 12 annually) would allow better perioperative outcomes. Such comparison has to stratify patients based on their preoperative comorbidities, the factors leading to the choice of operative technique, and their perioperative complications classified on Clavien-Dindo scale.
- To define what is the exact proportion of patients who undergo adrenalectomy using a laparoscopic approach in different centres/countries. Initial reports suggest that in recent years, laparoscopic adrenalectomy was performed in 78% of patients in Scandinavia [82] but maybe in as little as 35% in US [18]. This variability should be defined better and causes that lead to such differences addressed.

## Consensus statement

The ESES membership considered the information outlined in this manuscript and debated the issues raised during the ESES2019 meeting. This overall consensus provides guidance for the development of adrenal surgery in Europe in the next decade.

1. Minimum workload.

1.a. ESES recommends that laparoscopic adrenal surgery should be performed only in units with a predicted annual workload of 6 or more cases/year (level of evidence III/grade of recommendation B).

Though this number might appear surprisingly low, achieving this aim would bring significant restructuring of the current service provision for adrenal surgery. It is deemed to be a realistic and feasible goal and each national society should explore ways of addressing this issue.

1.b. Open adrenalectomy for high-risk patients (i.e. suspected or diagnosed adrenocortical cancer or phaeochromocytomas) should be offered only in units with a track record of involvement in the care of such patients and with a workload of minimum of 12 cases/year (level of evidence III/grade of recommendation B).

2. Choice of technique.

2.a. For the ‘novice’ adrenal surgeon, laparoscopic transabdominal surgery should be the first choice as it can be offered to the widest spectrum of adrenal conditions and it can be ‘mastered’ within 20–40 cases (level of evidence III/grade of recommendation C).

2.b. Retroperitoneoscopic surgery should be adopted by surgeons with previous experience in transabdominal adrenalectomy and with a high-volume practice so that sufficient eligible patients can be encountered within a reasonable time during their personal learning curve (level of evidence III/grade of recommendation C).

2.c. Robotic adrenal surgery should be implemented only in units with previous experience in robotic surgery, familiar with laparoscopic adrenalectomy and with large-volume practice. Robotic adrenalectomy is not a technique for the occasional adrenal surgeon with minimal previous personal experience (level of evidence V/grade of recommendation C).

3. Multidisciplinary input

Adrenal surgery should be offered only in centres with capacity for a valid multidisciplinary team consisting of oncologist, surgeon, endocrinologist, radiologist (level of evidence III/grade of recommendation B).

The complexity of medical care of many patients with adrenal pathology, the potential need for multi-specialty input in some adrenal operations (e.g. IVC involvement, simultaneous resections of small-volume metastatic disease), the need for accurate pathology reporting, and the benefits of oncological opinion regarding adjuvant or palliative chemotherapy should restrict adrenal surgery to medical institutions where such multidisciplinary input can be provided.

4. Role of referral centres

The governing principles for defining a referral centre should be based on (i). sufficient annual workload (> 12 cases/year), (ii) presence of an experienced multidisciplinary team and (iii) involvement of certified surgeons. Good clinical practice demands the need for clinical governance, the requirements of obtaining informed consent based on quoting personal incidence of untoward events (e.g. morbidity, conversion to open operation, intraoperative bleeding).

The increasing scrutiny of medical profession by governing bodies and patients should trigger a change in the current delivery of adrenal surgery. Each EU country will have to define the mechanism of establishing referral centres based on advice from national surgical and medical professional societies in collaboration with ESES and ENSAT. Because the variation in health care systems and mechanisms for reimbursement vary between countries, the models and process to reach such a change in practice is expected to differ between countries.

5. Data collection

Clinical information regarding adrenalectomies should be recorded prospectively and contribution to the established EUROCRINE and ENSAT databases is strongly encouraged.

6. Training of future adrenal surgeons

The ESES recommends that surgeons who want to develop an interest in adrenal surgery should seek training in reputable units, should aim to secure mentorship from an establish surgeon, should liaise with other specialties (endocrinology, radiology, pathology) to secure the development of an integrated multidisciplinary clinical service, and should monitor their personal learning curve through accurate data collection and audit.

Training of junior doctors with an interest in endocrine surgery and adrenal surgery is discussed in a separate document [83]

## References

[1] Song JH, Chaudhry FS, Mayo-Smith WW (2008). The incidental adrenal mass on CT: prevalence of adrenal disease in 1,049 consecutive adrenal masses in patients with no known malignancy. AJR Am J Roentgenol.

[2] Käyser SC, Dekkers T, Groenewoud HJ, van der Wilt GJ, Carel Bakx J, van der Wel MC, Hermus AR, Lenders JW, Deinum J (2016). Study heterogeneity and estimation of prevalence of primary aldosteronism: a systematic review and meta-regression analysis. J Clin Endocrinol Metab.

[3] Fassnacht M, Arlt W, Bancos I, Dralle H, Newell-Price J, Sahdev A, Tabarin A, Terzolo M, Tsagarakis S, Dekkers OM (2016). Management of adrenal incidentalomas: European society of endocrinology clinical practice guideline in collaboration with the European network for the study of adrenal tumors. Eur J Endocrinol.

[4] Etxabe J, Vasquez JA (1994). Morbidity and mortality in Cushing’s disease: an epidemiological approach. Clin Endocrinol.

[5] Valassi E, Santos A, Yaneva M, Tóth M, Strasburger CJ, Chanson P, Wass JA, Chabre O, Pfeifer M, Feelders RA, Tsagarakis S, Trainer PJ, Franz H, Zopf K, Zacharieva S, Lamberts SW, Tabarin A, Webb SM, ERCUSYN Study Group (2011). The European Registry on Cushing’s syndrome: 2-year experience. Baseline demographic and clinical characteristics. Eur J Endocrinol.

[6] Terzolo M, Reimondo G, Chiodini I, Castello R, Giordano R, Ciccarelli E, Limone P, Crivellaro C, Martinelli I, Montini M, Disoteo O, Ambrosi B, Lanzi R, Arosio M, Senni S, Balestrieri A, Solaroli E, Madeo B, De Giovanni R, Strollo F, Battista R, Scorsone A, Giagulli VA, Collura D, Scillitani A, Cozzi R, Faustini-Fustini M, Pia A, Rinaldi R, Allasino B, Peraga G, Tassone F, Garofalo P, Papini E, Borretta G (2012). Screening of Cushing’s syndrome in outpatients with type 2 diabetes: results of a prospective multicentric study in Italy. J Clin Endocrinol Metab.

[7] Dekkers OM, Horváth-Puhó E, Jørgensen JO, Cannegieter SC, Ehrenstein V, Vandenbroucke JP, Pereira AM, Sørensen HT (2013). Multisystem morbidity and mortality in Cushing’s syndrome: a cohort study. J Clin Endocrinol Metab.

[8] Plouin PF, Amar L, Chatellier G (2004). Trends in the prevalence of primary aldosteronism, aldosterone-producing adenomas, and surgically correctable aldosterone-dependent hypertension. Nephrol Dial Transplant.

[9] Jansen PM, Boomsma F, van den Meiracker AH, Dutch AI (2008). Aldosterone-to-renin ratio as a screening test for primary aldosteronism – the Dutch ARRAT Study. Neth J Med.

[10] Hannemann A, Wallaschofski H (2012). Prevalence of primary aldosteronism in patient’s cohorts and in population-based studies–a review of the current literature. Horm Metab Res.

[11] Björklund P, Backman S (2018). Epigenetics of pheochromocytoma and paraganglioma. Mol Cell Endocrinol.

[12] Eisenhofer G, Siegert G, Kotzerke J, Bornstein SR, Pacak K (2008). Current progress and future challenges in the biochemical diagnosis and treatment of pheochromocytomas and paragangliomas. Horm Metab Res.

[13] Lenders JW, Duh QY, Eisenhofer G (2014). Pheochromocytoma and paraganglioma: an endocrine society clinical practice guideline. J Clin Endocrinol Metab.

[14] Lo CY, Lam KY, Wat MS, Lam KS (2000). Adrenal pheochromocytoma remains a frequently overlooked diagnosis. Am J Surg.

[15] Rossitti HM, Soderkvist P, Gim O (2018). Extent of surgery for phaeochromocytoma in the genomic era. BJS.

[16] Mihai R (2015). Surgery for adrenocortical cancer – a systematic review. Br J Surg.

[17] Fassnacht M, Dekkers O, Else T, Baudin E, Berruti A, de Krijger RR, Haak HR, Mihai R, Assie G, Terzolo M (2018). European Society of Endocrinology Clinical Practice Guidelines on the management of adrenocortical carcinoma in adults, in collaboration with the European Network for the study of adrenal tumors. Eur J Endocrinol.

[18] Lindeman B, Hashimoto DA, Bababekov YJ, Stapleton SM, Chang DC, Hodin RA, Phitayakorn R (2018). Fifteen years of adrenalectomies: impact of specialty training and operative volume. Surgery.

[19] Park HS, Roman SA, Sosa JA (2009). Outcomes from 3144 adrenalectomies in the United States: which matters more, surgeon volume or specialty?. Arch Surg.

[20] Faiena I, Tabakin A, Leow J, Patel N, Modi PK, Salmasi AH, Chung BI, Chang SL, Singer EA (2017). Adrenalectomy for benign and malignant disease: utilization and outcomes by surgeon specialty and surgical approach from 2003-2013. Can J Urol.

[21] Sood A, Majumder K, Kachroo N, Sammon JD, Abdollah F, Schmid M, Hsu L, Jeong W, Meyer CP, Hanske J, Kalu R, Menon M, Trinh QD (2016). Adverse event rates, timing of complications, and the impact of specialty on outcomes following adrenal surgery: an analysis of 30-day outcome data from the American College of Surgeons National Surgical Quality Improvement Program (ACS-NSQIP). Urology..

[22] Monn MF, Calaway AC, Mellon MJ, Bahler CD, Sundaram CP, Boris RS (2015). Changing USA national trends for adrenalectomy: the influence of surgeon and technique. BJU Int.

[23] Villar JM, Moreno P, Ortega J, Bollo E, Ramírez CP, Muñoz N, Martínez C, Domínguez-Adame E, Sancho J, del Pino JM, Couselo JM, Carrión A, Candel M, Cáceres N, Octavio JM, Mateo F, Galán L, Ramia JM, Aguiló J, Herrera F (2010). Results of adrenal surgery. Data of a Spanish National Survey. Langenbeck's Arch Surg.

[24] Palazzo F, Dickinson A, Phillips B, Sahdev A, Bliss R, Rasheed A, Krukowski Z, Newell-Price J (2016). Adrenal surgery in England: better outcomes in high-volume practices. Clin Endocrinol.

[25] BAETS audit (https://www.baets.org.uk/wp-content/uploads/BAETS-Audit-National-Report-2017.pdf accessed on 7 July 2019)

[26] Kerkhofs TM, Verhoeven RH, Bonjer HJ, van Dijkum EJ, Vriens MR, De Vries J, Van Eijck CH, Bonsing BA, Van de Poll-Franse LV, Haak HR (2013). Dutch Adrenal Network. Surgery for adrenocortical carcinoma in The Netherlands: analysis of the national cancer registry data. Eur J Endocrinol.

[27] Simhan J, Smaldone MC, Canter DJ, Zhu F, Starkey R, Stitzenberg KB, Uzzo RG, Kutikov A (2012). Trends in regionalization of adrenalectomy to higher volume surgical centers. J Urol.

[28] Caiazzo R, Marciniak C, Lenne X, Clément G, Theis D, Ménégaux F, Sebag F, Brunaud L, Lifante JC, Mirallie E, Bruandet A, Pattou F. Adrenalectomy risk score: an original pre-operative surgical scoring system to reduce mortality and morbidity after adrenalectomy. 2019 (submitted) 10.1097/SLA.0000000000003526 31592809

[29] Greilsamer T, Nomine-Criqui C, Thy M, Ullmann T, Zarnegar R, Bresler L, Brunaud L. Robotic-assisted unilateral adrenalectomy: risk factors for perioperative complications in 303 consecutive patients. Surg Endosc 2019; 33(3):802-810. 10.1007/s00464-018-6346-2 29998394

[30] Walz MK, Alesina PF, Wenger FA, Deligiannis A, Szuczik E, Petersenn S, Ommer A, Groeben H, Peitgen K, Janssen OE, Philipp T, Neumann HP, Schmid KW, Mann K (2006). Posterior retroperitoneoscopic adrenalectomy--results of 560 procedures in 520 patients. Surgery..

[31] Iacconi P, Donatini G, Iacconi C, De Bartolomeis C, Cucinotta M, Puccini M, Miccoli P (2008). Unexpected histological findings of lesions diagnosed in the adrenal region in a series of 420 patients submitted to adrenal surgery. Review of our experience. J Endocrinol Investig.

[32] Coste T, Caiazzo R, Torres F, Vantyghem MC, Carnaille B, Do Cao C, Douillard C, Pattou F (2017). Laparoscopic adrenalectomy by transabdominal lateral approach: 20 years of experience. Surg Endosc.

[33] Heger P, Probst P, Hüttner FJ, Gooßen K, Proctor T, Müller-Stich BP, Strobel O, Büchler MW, Diener MK (2017). Evaluation of open and minimally invasive adrenalectomy: a systematic review and network meta-analysis. World J Surg.

[34] Lee CW, Salem AI, Schneider DF, Leverson GE, Tran TB, Poultsides GA, Postlewait LM, Maithel SK, Wang TS, Hatzaras I, Shenoy R, Phay JE, Shirley L, Fields RC, Jin LX, Pawlik TM, Prescott JD, Sicklick JK, Gad S, Yopp AC, Mansour JC, Duh QY, Seiser N, Solorzano CC, Kiernan CM, Votanopoulos KI, Levine EA, Weber SM (2017). Minimally invasive resection of adrenocortical carcinoma: a multi-institutional study of 201 patients. J Gastrointest Surg.

[35] Al-Otaibi KM (2012). Laparoscopic adrenalectomy: 10 years experience. Urol Ann.

[36] Wittayapairoch J, Jenwitheesuk K, Punchai S, Saeseow OT, Thanapaisal C, Paonariang K (2015). Laparoscopic adrenalectomy: 6 years experience in Srinagarind Hospital. J Med Assoc Thail.

[37] Popov Z, Jankulovski N, Stankov O, Stavridis S, Saidi S, Kuzmanoski M, Chipurovski I, Banev S, Krstevska B, Ivanovski O, Dimitrovski C (2015). Laparoscopic adrenalectomy: first single-center experience in the Balkans. Pril (Makedon Akad Nauk Umet Odd Med Nauki).

[38] Stefanidis D, Goldfarb M, Kercher KW, Hope WW, Richardson W, Fanelli RD (2013). SAGES guidelines for minimally invasive treatment of adrenal pathology. Surg Endosc.

[39] Hallfeldt KKJ, Mussack T, Trupka A, Hohenbleicher F, Schmidbauer S (2003). Laparoscopic lateral adrenalectomy versus open posterior adrenalectomy for the treatment of benign adrenal tumors. Surg Endosc.

[40] Thompson GB, Grant CS, van Heerden JA, Schlinkert RT, Young WF, Farley DR (1997). Laparoscopic versus open posterior adrenalectomy: a case-control study of 100 patients. Surgery..

[41] Brunt LM, Doherty GM, Norton JA, Soper NJ, Quasebarth MA, Moley JF (1996). Laparoscopic adrenalectomy compared to open adrenalectomy for benign adrenal neoplasms. J Am Coll Surg.

[42] Goitein D, David G, Mintz Y, Yoav M, Gross D, Reissman P (2004). Laparoscopic adrenalectomy: ascending the learning curve. Surg Endosc.

[43] Sommerey S, Foroghi Y, Chiapponi C, Baumbach SF, Hallfeldt KKJ, Ladurner R (2015). Laparoscopic adrenalectomy--10-year experience at a teaching hospital. Langenbeck's Arch Surg.

[44] Walz MK, Peitgen K, Krause U, Eigler FW (1995). Dorsal retroperitoneoscopic adrenalectomy--a new surgical technique. Zentralbl Chir.

[45] Vrielink OM, Engelsman AF, Hemmer PHJ, de Vries J, Vorselaars WMCM, Vriens MR (2018). Multicentre study evaluating the surgical learning curve for posterior retroperitoneoscopic adrenalectomy. Br J Surg.

[46] van Uitert A, d'Ancona FCH, Deinum J, Timmers HJLM, Langenhuijsen JF (2017). Evaluating the learning curve for retroperitoneoscopic adrenalectomy in a high-volume center for laparoscopic adrenal surgery. Surg Endosc.

[47] Piazza L, Caragliano P, Scardilli M, Sgroi AV, Marino G, Giannone G Laparoscopic robot-assisted right adrenalectomy and left ovariectomy (case reports). Chir Ital 51(6):465–466 10742897

[48] Brunaud L, Bresler L, Ayav A, Zarnegar R, Raphoz A-L, Levan T (2008). Robotic-assisted adrenalectomy: what advantages compared to lateral transperitoneal laparoscopic adrenalectomy?. Am J Surg.

[49] Agcaoglu O, Aliyev S, Karabulut K, Mitchell J, Siperstein A, Berber E (2012). Robotic versus laparoscopic resection of large adrenal tumors. Ann Surg Oncol.

[50] Agrusa A, Romano G, Navarra G, Conzo G, Pantuso G, Di Buono G (2017). Innovation in endocrine surgery: robotic versus laparoscopic adrenalectomy. Meta-analysis and systematic literature review. Oncotarget..

[51] Gaujoux S, Mihai R (2017). joint working group of ESES and ENSAT. European Society of Endocrine Surgeons (ESES) and European Network for the Study of Adrenal Tumours (ENSAT) recommendations for the surgical management of adrenocortical carcinoma. Br J Surg.

[52] Shahian DM, Normand S-LT (2003). The volume-outcome relationship: from Luft to Leapfrog. Ann Thorac Surg.

[53] Anderson KL, Thomas SM, Adam MA, Pontius LN, Stang MT, Scheri RP (2018). Each procedure matters: threshold for surgeon volume to minimize complications and decrease cost associated with adrenalectomy. Surgery.

[54] Morelli L, Tartaglia D, Bronzoni J, Palmeri M, Guadagni S, Di Franco G (2016). Robotic assisted versus pure laparoscopic surgery of the adrenal glands: a case-control study comparing surgical techniques. Langenbeck's Arch Surg.

[55] Tessier DJ, Iglesias R, Chapman WC, Kercher K, Matthews BD, Gorden DL (2009). Previously unreported high-grade complications of adrenalectomy. Surg Endosc.

[56] Assalia A, Gagner M (2004). Laparoscopic adrenalectomy. Br J Surg.

[57] Boylu U, Oommen M, Lee BR, Thomas R (2009). Laparoscopic adrenalectomy for large adrenal masses: pushing the envelope. J Endourol.

[58] Tsuru N, Suzuki K, Ushiyama T, Ozono S (2005). Laparoscopic adrenalectomy for large adrenal tumors. J Endourol.

[59] Ali JM, Liau S-S, Gunning K, Jah A, Huguet EL, Praseedom RK (2012). Laparoscopic adrenalectomy: auditing the 10 year experience of a single centre. Surgeon.

[60] Shen WT, Grogan R, Vriens M, Clark OH, Duh Q-Y (2010). One hundred two patients with pheochromocytoma treated at a single institution since the introduction of laparoscopic adrenalectomy. Arch Surg.

[61] Cheah WK, Clark OH, Horn JK, Siperstein AE, Duh Q-Y (2002). Laparoscopic adrenalectomy for pheochromocytoma. World J Surg.

[62] Li ML, Fitzgerald PA, Price DC, Norton JA (2001). Iatrogenic pheochromocytomatosis: a previously unreported result of laparoscopic adrenalectomy. Surgery.

[63] Conzo G, Musella M, Corcione F, De Palma M, Ferraro F, Palazzo A (2013). Laparoscopic adrenalectomy, a safe procedure for pheochromocytoma. A retrospective review of clinical series. Int J Surg.

[64] Conzo G, Tartaglia E, Gambardella C, Esposito D, Sciascia V, Mauriello C (2016). Minimally invasive approach for adrenal lesions: Systematic review of laparoscopic versus retroperitoneoscopic adrenalectomy and assessment of risk factors for complications. Int J Surg.

[65] Castillo OA, Rodríguez-Carlin A, López-Vallejo J, Borgna V (2014). Complications associated with laparoscopic adrenalectomy: description and standardized assessment. Actas Urol Esp.

[66] Erbil Y, Barbaros U, Sari S, Agcaoglu O, Salmaslioglu A, Ozarmagan S (2010). The effect of retroperitoneal fat mass on surgical outcomes in patients performing laparoscopic adrenalectomy: the effect of fat tissue in adrenalectomy. Surg Innov.

[67] Agha A, Iesalnieks I, Hornung M, Phillip W, Schreyer A, Jung M (2014). Laparoscopic trans- and retroperitoneal adrenal surgery for large tumors. J Minim Access Surg.

[68] Henry J-F, Sebag F, Iacobone M, Mirallie E (2002). Results of laparoscopic adrenalectomy for large and potentially malignant tumors. World J Surg.

[69] Miller BS, Ammori JB, Gauger PG, Broome JT, Hammer GD, Doherty GM (2010). Laparoscopic resection is inappropriate in patients with known or suspected adrenocortical carcinoma. World J Surg.

[70] Brix D, Allolio B, Fenske W, Agha A, Dralle H, Jurowich C (2010). Laparoscopic versus open adrenalectomy for adrenocortical carcinoma: surgical and oncologic outcome in 152 patients. Eur Urol.

[71] Donatini G, Caiazzo R, Do Cao C, Aubert S, Zerrweck C, El-Kathib Z, Gauthier T, Leteurtre E, Wemeau JL, Vantyghem MC, Carnaille B, Pattou F (2014). Long-term survival after adrenalectomy for stage I/II adrenocortical carcinoma (ACC): a retrospective comparative cohort study of laparoscopic versus open approach. Ann Surg Oncol.

[72] Lombardi CP, Raffaelli M, Boniardi M, De Toma G, Marzano LA, Miccoli P (2012). Adrenocortical carcinoma: effect of hospital volume on patient outcome. Langenbeck's Arch Surg.

[73] Ludwig WW, Gorin MA, Pierorazio PM, Allaf ME (2017). Frontiers in robot-assisted retroperitoneal oncological surgery. Nat Rev Urol.

[74] Morche J, Mathes T, Pieper D (2016). Relationship between surgeon volume and outcomes: a systematic review of systematic reviews. Syst Rev.

[75] Al-Qurayshi Z, Robins R, Buell J, Kandil E (2016). Surgeon volume impact on outcomes and cost of adrenal surgeries. Eur J Surg Oncol.

[76] Kazaure HS, Roman SA, Sosa JA (2011). Adrenalectomy in older Americans has increased morbidity and mortality: an analysis of 6,416 patients. Ann Surg Oncol.

[77] Winter JM (2006). Thirty robotic adrenalectomies: a single institution’s experience. Surg Endosc.

[78] Economopoulos KP (2017). Laparoscopic versus robotic adrenalectomy: a comprehensive meta-analysis. Int J Surg.

[79] Feng Z, Feng MP, Feng DP, Rice MJ, Solorzano CC (2018). A cost-conscious approach to robotic adrenalectomy. J Robot Surg.

[80] Nomine-Criqui C, Germain A, Ayav A, Bresler L, Brunaud L (2017). Robot-assisted adrenalectomy: indications and drawbacks. Updat Surg.

[81] Arora S, Rogers CG, Menon M (2018). Re: Each procedure matters: threshold for surgeon volume to minimize complications and decrease cost associated with adrenalectomy. Surgery.

[82] Thompson LH, Nordenström E, Almquist M, Jacobsson H, Bergenfelz A (2017). Risk factors for complications after adrenalectomy: results from a comprehensive national database. Langenbeck's Arch Surg.

[83] Gimm O, Barczyński M, Mihai R, Raffaelli M. Training in endocrine surgery . Lang Arch Surgery 2019 (submitted) 10.1007/s00423-019-01828-4 PMC6935392 31701231

